# The testosterone paradox in asthma

**DOI:** 10.3389/falgy.2026.1740307

**Published:** 2026-04-23

**Authors:** Ynuk Bossé

**Affiliations:** Department of Medicine, Institut Universitaire de Cardiologie et de Pneumologie de Québec (IUCPQ) – Université Laval, Quebec, QC, Canada

**Keywords:** animal model, asthma, hyperresponsiveness, methacholine, respiratory mechanics

## Abstract

Asthma and an excessive response to inhaled methacholine, commonly called hyperresponsiveness, are intimately linked. Therefore, factors increasing the methacholine response are expected to contribute to asthma as well. However, this is clearly not the case for testosterone, the predominant sex hormone in males. While testosterone increases the methacholine response, it is rather protective in asthma and experimental asthma. These incompatible observations are referred herein as the ‘testosterone paradox’, and the chase is now on to unravel its underlying mechanisms.

## Introduction

Asthma is a lung disorder affecting an estimated 260 million people worldwide and accounting for more than 400 thousands premature deaths annually (1). The etiology and the pathogenesis of asthma are highly heterogeneous (2). Its symptoms are also typically fluctuating over time and coincide with exposures to offending environmental triggers, such as infectious pathogens, pollutants, allergens, cold air, and specific classes of drugs. Even though the mainstay treatments for asthma (*i.e.*, inhaled corticosteroids and *β*_2_-adrenoceptor agonists) are mimetics of hormones predominantly secreted by remote organs (*i.e.*, cortisol and epinephrine from adrenals), the endocrine regulation of asthma is currently overlooked (3). The present perspective focuses on the testosterone paradox, which refers to puzzling observations showing that while testosterone is protecting asthma, it is also seemingly contributing to one of its defining features.

### Hyperresponsiveness

Hyperresponsiveness is a defining feature of asthma (4). It is generally described as an exaggerated decline in lung function in response to a given challenge, such as a bout of physical exercise or the inhalation of a specific trigger. Measuring the response to these challenges involves sophisticated testing in laboratories of respiratory physiology that are still evolving today (5, 6). These test results are also thought to reflect the response that is naturally occurring when asthmatic patients are exposed to their respective offending triggers. Hyperresponsiveness is thus considered a feature that is significantly contributing to, if not the main driver of, asthma symptoms.

**Figure 1:**
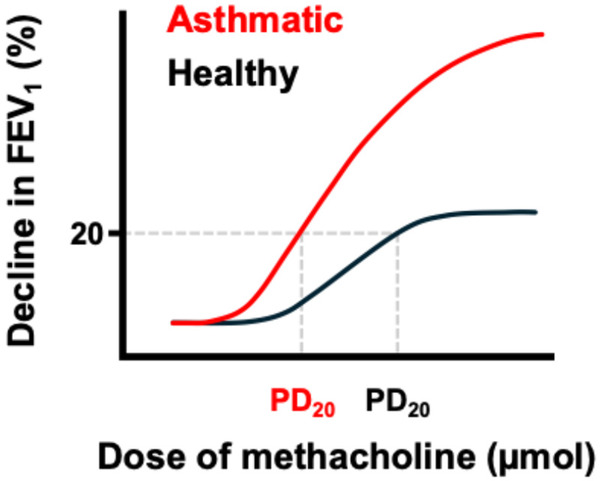
Measuring the methacholine response. The methacholine response in humans is typically monitored by measuring the decline in lung function during a challenge with incremental doses of inhaled methacholine. More precisely, the lung function that is being measured is called the forced expiratory volume in 1 s (FEV_1_), which is essentially the maximal amount of air that can be expelled by the subject during the first second of a forced expiration beginning from fully inflated lungs. Two dose-response curves were sketched on this illustration for comparing a normal response in an healthy individual (black) to an excessive response in an asthmatic individual (red). An excessive response is characterized by a leftward shift of the curve (an increased sensitivity) and an upward shift of the curve (an increased maximal response). In clinics, hyperresponsiveness is more specifically confirmed when the provocative dose causing a 20% decline in FEV_1_ (PD_20_) is inferior to 0.5 µmol. Note in the illustration how the PD_20_ of the asthmatic patient is lower.

One popular test to evaluate the degree of responsiveness involves the inhalation of methacholine (Figure 1), a molecule that is directly activating the contraction of the airway smooth muscle. The protocol for methacholine testing has been standardized and guidelines have been published (5). Hyperresponsiveness to inhaled methacholine implies that there are defects in the lungs that amplify the decline in lung function upon contraction of the airway smooth muscle. The test is particularly useful in clinics because of its strong negative predictive value, meaning that, if a patient with respiratory symptoms is not hyperresponsive to methacholine, the diagnosis is unlikely asthma (7). Debating against the underlying causes of methacholine hyperresponsiveness in asthma would be like debating against the underlying causes of asthma itself, as the methacholine challenge is merely a test that was created for guiding its diagnostics. Therefore, asthma and methacholine hyperresponsiveness are not only intimately linked but they supposedly stem from the very same fabrics (8, 9). Factors increasing the methacholine response are thus expected to contribute to asthma as well. However, this is not the case for testosterone.

### The testosterone paradox

The protective role of testosterone in human asthma is inferred mainly from indirect evidence. First, there is a strong and universal interaction between sex and age in the prevalence of asthma (10–12). Males are more affected than females during childhood, but then male prevalence plummets during adolescence when their levels of testosterone rise (10–12). The prevalence of asthma remains lower in males thereafter (10–12), which also coincides with circulating levels of testosterone that remain higher than females throughout adulthood (13). Second, there are many reports of positive associations between androgen levels and favorable asthma outcomes (14–20). For example, circulating levels of testosterone are inversely related to current asthma prevalence in both males and females (16). In fact, it was estimated that every doubling concentration of circulating testosterone reduces asthma prevalence by about 10% (16). Testosterone also positively correlates with better lung function in asthmatic patients of both sexes, with an estimated gain of 300 mL in forced expiratory volume in 1 s (FEV_1_) for every doubling cocentration of circulating testosterone (16). Although interesting, one caveat to consider in these human studies is that corticosteroids, one of the mainstay therapy for asthma, decrease testosterone levels (20–25). Since patients with more severe asthma are typically treated with greater doses of corticosteroids, it implies that these associations between testosterone levels and asthma outcomes may be epiphenomena.

I am only aware of one early study that has tested the effect of testosterone on asthma outcomes in humans (26). The patients, who were all females and severe asthmatics, underwent short courses of treatment with either a daily tablet of 10 mg during 5 days or a single injection of 10.5 mg combined with 500 IU of chorionic gonadotrophin. The effects were quite remarkable, with most patients improving considerably and many showing no more asthmatic attacks (26). The injection of testosterone was even successful for acutely relieving patients from *status asthmaticus* (26). Testosterone replacement therapy was also successfully used in asthmatic men to reverse side effects of corticosteroids, including osteopenia, fat gain, and muscle wasting (27, 28). Unfortunately, these studies did not assess asthma outcomes, apart from reporting an overall improvement in well-being.

Dehydroepiandrosterone, another androgen less masculinizing than testosterone, has also been tested on asthma outcomes in humans (29, 30). Compare to placebo, a once-daily nebulized dose of 70 mg of dehydroepiandrosterone for 6 weeks was shown to significantly improve scores on the Asthma Control Questionnaire (ACQ) without affecting lung function in moderate-to-severe asthmatic patients who remained uncontrolled with low-dose inhaled corticosteroids plus a long-acting *β*_2_-agonist (29). Twice-daily oral doses of 100 mg of dehydroepiandrosterone also improved lung function in premenopausal women with mild-to-moderate asthma with low baseline levels of endogenous dehydroepiandrosterone (19).

In murine models, it is also generally recognized that females exhibit a greater susceptibility for the development of experimental asthma (31–43). Again, this is suggestive of a protective role of testosterone in experimental asthma. More directly, several studies have investigated the effect of testosterone on several facets of inflammation in different murine models of asthma. The findings are quite unanimous. Testosterone inhibits inflammation in experimental asthma (44–54). This has been attributed to several mechanisms, including the suppression of IL-4 and IL-17A production (48), a negative control over group 2 innate lymphoid cells (ILC2s) (45, 46, the promotion of suppressive functions in regulatory T cells (Tregs) (51), direct action on CD4^+^ T cells such as the downregulation of cytokine production (50), the differentiation of T-helper (Th) lymphocytes into Th1 cells (54), the inhibition of Th2 cell differentiation (52), and the inhibition of glutaminolysis and the suppression Th17 cells (53). Therefore, compelling evidence, in both mice and humans, suggest that testosterone is protecting against asthma.

Paradoxically, convincing experimental studies in healthy mice (*i.e.*, without experimental asthma) demonstrated that males are more responsive to nebulized methacholine than females (43, 55–60). This sexual difference is also not small. In order to achieve approximately the same response, the concentration of nebulized methacholine needs to be two times greater in females than males (57, 58, 61).

This sexual difference in the methacholine response in healthy mice also seems to be driven by testosterone. This is first supported by studies showing a decreased methacholine response in males post-orchiectomy (55, 56, 62). In fact, orchiectomized males exhibit a response similar to the one observed in females (55, 56). It is also supported by studies supplementing mice with testosterone, showing that testosterone increases the methacholine response in both females and orchiectomized males (55, 56). In fact, females supplemented with testosterone reaches a methacholine response comparable to the one observed in males (55, 56). The underlying mechanisms were also investigated and it seems to rely on intact vagal nerves. Indeed, bilateral vagotomy (*i.e.*, severing vagal nerves running on either side of the trachea) prevented the amplifying effect of testosterone on the methacholine response (56). How testosterone acts through the vagal nerves to potentiate the methacholine response is still unknown.

In mice with experimental asthma, the sex difference in the methacholine response is not as clear as in healthy mice. In models induced by inflammatory triggers (*e.g.*, house dust mite, ovalbumin, *etc*.), the results are mixed. Some investigations suggested an increased response in females (48, 53), others in males (43), and others reported no sexual differences (37, 38). The direct effect of testosterone on the methacholine response in mice with experimental asthma is also not clear. Previous investigations have suggested an attenuating effect of testosterone on hyperresponsiveness (49, 52, 53, 63). This finding is not universal though (54). The final effect of testosterone on hyperresponsiveness in these murine models of asthma probably comes down to some sort of balance that is discussed in the next paragraphs. In other models of experimental asthma caused by alterations in the airway smooth muscle, such as in the AJ mice (64) or in transgenic mice with enlarged airway smooth muscle, such as in the model of Kim and coworkers (65), sexual differences and the direct effect of testosterone on the methacholine response have yet to be studied. These studies would provide enlightening insights.

### Hints reconciling the testosterone paradox

Perhaps one way forward is to recognize that, although responsiveness and hyperresponsiveness to methacholine are inevitably intertwined because they are both actuated by smooth muscle activation, their genesis can be very different (63). So that the fabrics of the methacholine response might not be the same as the ones liable for hyperresponsiveness and asthma. The normal response in healthy individuals is obviously driven by the contraction of the airway smooth muscle. Perhaps that, somehow, testosterone potentiates this initial smooth muscle-mediated airway constriction and consequently increases the methacholine response, like it does in healthy mice (55, 56, 62). The same events are likely operational in experimental asthma as well, and are thus expected to worsen hyperresponsiveness. Yet, the scenario is much more complicated in asthma because the primary causes of hyperresponsiveness may have nothing to do with an exaggerated contraction of the airway smooth muscle (66–68).

The manifestation of hyperresponsiveness in experimental asthma more likely originates from an adverse synergistic interaction between inflammation and the contraction of the airway smooth muscle (59, 69). For example, airway wall edema, together with the accumulation of inflammatory, exudative, and mucosal fluids, increases luminal airway narrowing caused by any degree of airway smooth muscle contraction (69). Additionally, edema and the accumulation of fluids increase the propensity for small airway narrowing heterogeneity and closure, which can trigger important decline in lung function during methacholine testing. In fact, many considered airway narrowing heterogeneity and closure as the major causes of hyperresponsiveness in asthma (70–76) and experimental asthma (66, 77–81). Hyperresponsiveness to methacholine in asthma is therefore thought to be mainly driven by inflammation, even though the initial airway constriction is, as in non-asthmatics, mediated by the contraction of the smooth muscle.

Since testosterone markedly inhibits inflammation (as outlined above), it is expected to block the adverse synergistic interaction between inflammation and smooth muscle contraction that is conducive to hyperresponsiveness. The final effect of testosterone on hyperresponsiveness is therefore difficult to predict. Imagine a scenario where hyperresponsiveness would be made out of two fabrics. The final effect of a molecular mediator increasing fabric 1 but decreasing fabric 2 would be uncertain. The outcome would depend on the predominating fabric, and the magnitude by which each fabric is affected by the molecular mediator. Perhaps this is the case for testosterone. It may potentiate airway constriction mediated by smooth muscle contraction (let say fabric 1) while decreasing inflammation (let say fabric 2). The overall tilt of such a balance, between two fabrics moving in opposite directions, would probably exert only a minor (positive or negative) influence on hyperresponsiveness. This may explain why studies investigating the effect of testosterone on hyperresponsiveness in experimental asthma have been inconsistent (48, 49, 52–54).

This scenario would also reconcile the testosterone paradox. The protective effect of testosterone in asthma would then be attributed exclusively to its anti-inflammatory effect. The potentiating effect of testosterone on smooth muscle contraction would probably be uneventful in asthma. This is because the dampened inflammation would be counterbalancing it by mitigating the adverse synergistic interaction between inflammation and airway smooth muscle contraction.

Another related scenario, perhaps less popular but that I strongly advocate, would be that airway constriction caused by a naturally occurring smooth muscle contraction can sometimes protect against hyperresponsiveness and asthma (82). So molecular mediators increasing the methacholine response, such as testosterone, may sometimes prevent hyperresponsiveness. I understand that it may sound counterintuitive. A lot of people visualize the smooth muscle as the culprit. So how it can inversely protect again hyperresponsiveness?

The idea was born from studying ‘force adaptation’, a process whereby the contractile capacity of the airway smooth muscle is gradually increasing during a sustained contraction (83–86). As expected, force adaptation was shown to increase the methacholine response in both healthy mice (57, 58, 87, 88) and humans (89, 90). However, when it was tested in mice with experimental asthma, it decreased hyperresponsiveness (88), which sounds a lot like the paradoxical effect of testosterone. The proposed underlying mechanism by which force adaptation protects against hyperresponsiveness involves the stiffening of the lung tissue. More specifically, when the tens of thousands of airways within the lungs are constricted simultaneously, it is thought to increase the force of interdependence between them, which would then inhibit each other from further narrowing (91). It should also compel the lungs to work more homogeneously and thereby prevent small airway narrowing heterogeneity and closure (88), which, as aforementioned, are major causes of hyperresponsiveness in asthma (70–76) and experimental asthma (66, 77–81).

Again, in this scenario, fabrics 1 and 2 would be smooth muscle contraction and inflammation, respectively. Yet, fabric 1 would already be activated before the methacholine challenge and also at the right location (*i.e.*, throughout the lungs including in small peripheral airways) instead of being activated patchily and predominantly in large airways, such as during an inhaled delivery of methacholine (92). Fabric 2 would still be expected to increase luminal airway narrowing; *i.e.*, airway wall edema and accumulation of fluids would amplify luminal narrowing for any degree of smooth muscle shortening. However, the pre-activation of fabric 1 at the right location would prevent two major causes of hyperresponsiveness, namely narrowing heterogeneity and closure, which would presumptively tilt the overall balance toward a blunted hyperresponsiveness.

This other scenario would also reconcile the testosterone paradox. In this scenario, however, the protective effect of testosterone in asthma would rely on a two-birds-with-one-stone phenomenon, increasing contraction and decreasing inflammation. More studies will obviously be needed.

## Conclusion

While a compendium of human and mouse studies suggests that testosterone is protective against asthma, convincing experimental studies on mice demonstrate that it increases the methacholine response in healthy subjects. These observations obviously sound paradoxical for experts in respiratory research and medicine, because factors increasing the methacholine response typically promote asthma. In this perspective, I have coined these seemingly incompatible observations the testosterone paradox. I have also outlined different scenarios, hinted from recent findings in experimental studies, that tentatively explain the testosterone paradox. However, at this time, my arguments are largely speculative. Further studies in both humans and animals will provide definitive answers. The hunt in now on for unravelling the mechanisms underlying the testosterone paradox. I think scientists participating to this hunt lie in the way of promising discoveries that will not only transform the field of respiratory medicine but will also transcend into other disciplines, including endocrinology.
